# The Role of Students’ Beliefs When Critically Reasoning From Multiple Contradictory Sources of Information in Performance Assessments

**DOI:** 10.3389/fpsyg.2020.02192

**Published:** 2020-09-11

**Authors:** Olga Zlatkin-Troitschanskaia, Klaus Beck, Jennifer Fischer, Dominik Braunheim, Susanne Schmidt, Richard J. Shavelson

**Affiliations:** ^1^Department of Business and Economics Education, Johannes Gutenberg University Mainz, Mainz, Germany; ^2^Graduate School of Education, Stanford University, Palo Alto, CA, United States

**Keywords:** critical reasoning, multiple source use, reasoning profiles, performance assessment, domain-specific beliefs, decision-making, cognitive interview protocols, criteria-driven online search

## Abstract

*Critical reasoning* (CR) when confronted with contradictory information from multiple sources is a crucial ability in a knowledge-based society and digital world. Using information without critically reflecting on the content and its quality may lead to the acceptance of information based on unwarranted claims. Previous personal beliefs are assumed to play a decisive role when it comes to critically differentiating between assertions and claims and warranted knowledge and facts. The role of generic epistemic beliefs on critical stance and attitude in reflectively dealing with information is well researched. Relatively few studies however, have been conducted on the influence of *domain-specific beliefs*, *i.e., beliefs in relation to specific content encountered in a piece of information or task, on the reasoning process*, and on how these beliefs may affect decision-making processes. This study focuses on students’ *task- and topic-related beliefs* that may influence their reasoning when dealing with multiple and partly contradictory sources of information. To validly assess CR among university students, we used a newly developed computer-based *performance assessment* in which the students were confronted with an authentic task which contains theoretically defined psychological stimuli for measuring CR. To investigate the particular role of task- and topic-related beliefs on CR, a purposeful sample of 30 university students took part in a performance assessment and then were interviewed immediately afterward. In the semi-structured cognitive interviews, the participants’ task-related beliefs were assessed. Based on qualitative analyses of the interview transcripts, three distinct *profiles of decision-making* among students have been identified. More specifically, the different types of students’ beliefs and attitudes derived from the cognitive interview data suggest their influence on information processing, reasoning approaches and decision-making. The results indicated that the students’ beliefs had an influence on their selection, critical evaluation and use of information as well as on their reasoning processes and final decisions.

## Research Background and Study Objectives

Critical reasoning (CR) when confronted with contradictory information from multiple sources is a crucial ability in a knowledge-based society and digital world (Brooks, 2016; Newman and Beetham, 2017; Wineburg and McGrew, 2017). The Internet presents a flood of complex, potentially conflicting, and competing information on one and the same issue. To build a dependable and coherent knowledge base and to develop sophisticated (domain-specific and generic) attitudes and an analytical, reflective stance, students must be able to select and critically evaluate, analyze, synthesize, and integrate incoherent, fragmented, and biased information.

Students’ mental CR strategies may likely be insufficient for what is demanded for understanding heterogeneous information and, what is more, for effective and productive participation in a complex information environment (for a meta-study, see Huber and Kuncel, 2016, for university students, see McGrew et al., 2018; Wineburg et al., 2018; Hahnel et al., 2019; Münchow et al., 2019; Shavelson et al., 2019; Zlatkin-Troitschanskaia et al., 2019b). As a coping strategy, they may choose to reduce complexity by various means, for instance, by using cognitive heuristics, preferring simplified forms of information presentation, or relying on sources without verification, which can be exploited for manipulation (Walthen and Burkell, 2002; Metzger, 2007; Horstmann et al., 2009; Metzger et al., 2010).

In addition, certain information representations may be (sub)consciously preferred not for their informational but for their entertainment value, their elicitation of certain affects, or their engagement properties (Maurer et al., 2018, 2020). Based on students’ previous media experience, knowledge, and expectations, they may have learned to assume that certain types of media representations are more trustworthy than others (McGrew et al., 2017). They may follow a heuristic that similar media representations offer similarly reliable evidence, without considering the communicative context, communicator’s intentions, and possibilities of becoming a victim of manipulation. This is particularly the case when it comes to online information channels (Metzger et al., 2010; Ciampaglia, 2018).

As some current studies indicate, students who habitually avoid information that contradicts their beliefs may easily miss important content and fall prey to biased information [see Section “State of Research on Beliefs and Their Impact on (Online) Information Processing”]. Using information without critically reflecting on the content and its quality may lead to the acceptance of information based on unwarranted claims. Deficits in due critical evaluation arise most likely because of shallow processing or insufficient reasoning and may occur subconsciously (Stanovich, 2003, 2016).

Insufficient reasoning can be amplified when information on a topic is distorted or counterfactual and when students do not recognize biased or false information and use it to build knowledge. As a result, learners may neglect complex, academically warranted knowledge and rely more on lower-quality information that is consistent with their beliefs and biases and that is easier to comprehend (Hahnel et al., 2019; Schoor et al., 2019). The internalization of this biased information may subsequently affect learning by acting to inhibit or distort more advanced information processing and knowledge acquisition (List and Alexander, 2017, 2018).

Theoretically, previous personal beliefs are assumed to play a very decisive role when it comes to critically differentiating between assertions and claims on the one hand and warranted knowledge and facts on the other hand. Rather, the role of generic epistemic beliefs on critical stance and attitude in reflectively dealing with information is well researched [see Section “State of Research on Beliefs and Their Impact on (Online) Information Processing”]. Relatively few studies have been conducted on the influence of *domain-specific beliefs, i.e., beliefs in relation to specific content encountered in a piece of information or task*, on the reasoning process. Beliefs of this kind are usually measured using psychological scales, but without insight into the reasoning process and how these beliefs may affect the information-processing and decision-making processes [for an overview of current research, see Section “State of Research on Beliefs and Their Impact on (Online) Information Processing”].

With our study, we aim to contribute to this research desideratum. This study focuses on students’ *task- and topic-related beliefs* that may influence their reasoning when dealing with multiple and partly contradictory sources of information. To validly assess CR among university students, we used a newly developed computer-based performance assessment of learning in which the students are confronted with an authentic task which contains theoretically defined psychological stimuli for measuring CR (for details, see Section “Assessment Frameworks for Measuring Critical Reasoning”) in accordance with our construct definition (see Section “Critically Reasoning from Multiple Sources of Information”; Shavelson et al., 2019; Zlatkin-Troitschanskaia et al.,2019b).

To investigate the particular role of task- and topic-related beliefs on CR, a purposeful sample of 30 university students from the overall sample of the overarching German iPAL study took part in a performance assessment and then were interviewed immediately afterward (for details, see Sections “A Study on Performance Assessments of Higher Education Students’ CR” and “Materials and Methods”). In the semi-structured cognitive interviews, the task-related beliefs of the participants were elicited and then qualitatively analyzed (see Section “Cognitive Interviews and Qualitative Analyses”). The cognitive interview transcripts were examined in order to address the two overarching questions *(i) how do students’ beliefs influence their selection, evaluation and use of information as well as their subsequent reasoning and decision-making? and (ii) how do students’ beliefs change as they progress through the task and encounter multiple new information sources along the way.* Based on qualitative analyses (Strauss and Corbin, 1998; for details, see “Materials and Methods”), different profiles of participants have been identified, which can be distinguished by different personal characteristics such as the level of prior knowledge.

In this paper, we present our theoretical and empirical analyses to address these two questions (see Section “Results”). The study results – despite the necessary limitations (see Section “Limitations and Implication for Future Research”) – lead to a valuable specification of theoretical assumptions for further empirical research in this highly relevant but under-researched field (see Section “Summary and Interpretation of Results”).

## State of Research on Beliefs and Their Impact on (Online) Information Processing

For a systematic analysis of the state of research, we conducted a criteria-driven online search. Based on expert interviews, we determined a set of keywords and conducted the search on the ERIC database and Google Scholar for the period 2009–2020. The stepwise search using keywords related to online information processing and critical reasoning among university students resulted in 56 eligible studies. The review of the abstracts showed that students’ beliefs were assessed and analyzed in 15 studies. The essential results of these studies are summarized in the following overview (see Table 1).

**TABLE 1:** Overview of recent studies on beliefs and their impact on information processing.

Authors	Study	Focus of analysis	Study design	Sample
(1) Bråten et al., 2011	Trust and mistrust when students read multiple information sources about climate change	Evaluation of source information	Demographic information sheet, Topic knowledge questionnaire, texts and trustworthiness questionnaire	128 undergraduate students (80.2% female) from a university in southeast Norway
(2) Chiu et al., 2013	Internet-specific epistemic beliefs and self-regulated learning in online searches for academic information	Internet-specific epistemic beliefs	Exploratory factor analysis, confirmatory analysis and the hypothesized model	748 male and female university students in Taiwan
(3) Chiu et al., 2015	Testing measurement invariance and latent mean differences across gender groups in college students’ Internet-specific epistemic beliefs.	Internet-specific epistemic beliefs	Internet-specific epistemic beliefs questionnaire	735 male and female university students in Taiwan
(4) Hsu et al., 2014	Epistemic beliefs, online search strategies, and behavioral patterns while exploring socioscientific issues	Scientific-epistemic beliefs (SEB)	SEB questionnaire, Online Information Searching Strategies Inventory, screen-capture videos, sequential analysis	42 undergraduate and graduate students in Taiwan
(5) Johnson et al., 2016	Students’ approaches to the evaluation of digital information: Insights from their trust judgments	Trust judgements	55 5-point Likert-scale statements, questions about their disposition to trust and their health status	531 1st-year and 3rd year-students
(6) Kahne and Bowyer, 2017	Educating for democracy in a partisan age: confronting the challenges of motivated reasoning and misinformation	Digital media and motivated reasoning	Survey about students’ online activity and political participation	2101 participants (17–25 years old)
(7) Kammerer and Gerjets, 2012	Effects of search interface and Internet-specific epistemic beliefs on source evaluations during Web search for medical information	Internet-specific epistemic beliefs	Web search, source evaluations, search interface design, eye-tracking	80 university students from different majors at a German university
(8) Kienhues et al., 2011	Dealing with conflicting or consistent medical information on the web	Epistemic beliefs	Pretest–posttest experimental design: an intervention group (website with conflicting contents), another intervention group (website with consistent contents) and a no-intervention group (control group, no web search)	100 mostly (84%) female students attending a German university
(9) Lucassen and Schraagen, 2011	Factual accuracy and trust in information: The role of expertise	Trust judgements; source, semantic, surface-model	Online questionnaire; novice–expert-design	657 participants (recruited in different online forums)
(10) Mason et al., 2010	Searching the Web to learn about a controversial topic: are students epistemically active?	Epistemic metacognition	Online information searching; think-aloud procedure during the search; two measures for verbal and visuospatial memory capacity; writing an essay	46 students from an university in northern Italy
(11) Mason et al., 2014	Reading information about a scientific phenomenon on webpages varying for reliability: an eye-movement analysis	Source evaluation; eye movements; Internet reading	Eye-tracking; prior knowledge questions, complete the Connotative Aspects of Epistemological Beliefs and then read the 4 webpages to get information for writing a report	49 female undergraduate students from the faculty of psychology, public university in north-eastern Italy
(12) van Strien et al., 2012b	Do prior attitudes influence epistemic cognition while reading conflicting information?	Epistemic cognition	Reading a number of pre-selected texts on climate change; thinking aloud; writing a short essay	98 students from a Dutch school for pre-university education; 25 students in the follow-up study
(13) van Strien et al., 2014	Dealing with conflicting information from multiple nonlinear texts: Effects of prior attitudes	Prior attitudes; evaluating conflicting information	Reading and writing task (essays were scored on the perspective taken and the origin of information)	61 students in pre-university education in the Netherlands
(14) van Strien et al., 2016	How attitude strength biases information processing and evaluation on the Internet	Prior attitudes; attitude strength; source evaluation	Online questionnaire; eye-tracking; writing an essay; computer-based questionnaire	79 students (56 female) from a German university
(15) Ulyshen et al., 2015	Understanding the connection between epistemic beliefs and Internet searching	Epistemic beliefs	An ill-structured task using the Google search engine; the revised Cognitive Flexibility Inventory, prior content knowledge test, verbal comprehension test, complexity of learning strategies (think aloud procedure) & retrospective interview	53 undergraduate students from a Midwestern University

About half of these 15 studies focus explicitly on the relation between beliefs and (online) information processing (see Table 1), while critical reasoning was only implicitly addressed. Despite this narrow research focus, all studies indicate a clear connection between *epistemic beliefs* and the approach to (online) information processing, especially regarding *judgment* of information sources and their content. Well-developed and more advanced epistemic beliefs positively influenced the quality of students’ information processing.

Ulyshen et al. (2015) provided one of the few studies specifically investigating the relation between *general epistemic beliefs* and *Internet search behavior*. Using participants’ think-aloud protocols they investigated the impact of students’ task-related epistemic beliefs on their information processing. The results indicate a positive impact of well-developed epistemic beliefs on evaluating the quality and credibility of information.

Chiu et al. (2013) used a questionnaire to investigate the relation between university students’ *Internet-specific epistemic beliefs* and Internet search behavior. The authors identified four dimensions of beliefs: *certainty, simplicity, source*, and *justification of Internet-based knowledge*. The results indicate a positive association between Internet searching and *justification*, and a negative association with *simplicity and source*. In a follow-up study, Chiu et al. (2015) examined gender differences in students’ Internet-specific epistemic beliefs, indicating a gender gap in *certainty and simplicity*, and revealing more perceived *uncertainty* and *complexity* among females compared to males.

Mason et al. (2010) specifically focused on students’ *Internet search* when working on *academic tasks dealing with a controversial topic*, and in relation to *epistemic metacognition*, which was defined as students’ ability to spontaneously reflect on the accessed information. In addition, they examined the relationship between personal characteristics and prior topic-related knowledge. Test participants were asked to *think aloud during their Internet search*. Qualitative and quantitative analyses revealed diverse epistemic metacognitions among all study participants, but to different extents and levels. No correlation between epistemic beliefs and prior knowledge was identified. Overall, two patterns of epistemic metacognition were determined that significantly affected students’ Internet search. Students who spontaneously generated more sophisticated reflections about the sources and the information provided outperformed students who were at a lower epistemic level.

In an experimental study with an intervention-control group design (the intervention aiming at improving *medicine-specific epistemic beliefs*), Kienhues et al. (2011) focused on the relationship between *processing conflicting versus consistent (medical) information on the Internet and topic-related and medicine-specific epistemic beliefs*. The intervention groups differed in both their topic-related and medicine-specific epistemic beliefs, and were more advanced compared to the control group.

van Strien et al. (2016) examined the influence of attitude strength on the processing and evaluation of sources of information on the Web. In an eye-tracking study, university students received information from pre-selected websites from different sources on a controversial topic. Participants who felt strongly about the topic did not consider websites with attitude-inconsistent content for as long and did not rate the credibility of this information as highly as students with less strongly established prior attitudes. The participants with strong prior attitudes also included more attitude-consistent information in an essay task than participants with weaker prior attitudes. Thus, differences in prior attitudes bias the evaluation and processing of information in different ways. Even though students were not fully biased during initial information processing, they were so when evaluating the information and presenting it in an essay task.

Similar biases were found in a study by Bråten et al. (2011), who examined how undergraduates judged the trustworthiness of information sources on a controversial topic. Students judged information differently depending on the *sources* (e.g., textbooks, official documents, newspapers). In addition, students with limited *topic-specific knowledge* were inclined to trust less trustworthy sources. Lucassen and Schraagen (2011) show similar results in terms of relation with *domain-specific knowledge and source expertise*.

Following the assumption that students spontaneously engage in *epistemic cognition* when processing conflicting scientific information, van Strien et al. (2012b) examined how this epistemic cognition is related to students’ actual beliefs. In addition, the interplay of students’ epistemic beliefs and prior attitudes when encountering conflicting and partly attitude-inconsistent information on a controversial socio-scientific topic was studied using think-aloud methods. The results indicated that students’ difficulties in adequately evaluating diverse and conflicting information do not correlate with their *prior epistemic beliefs*. These beliefs might be developing from *naïve to sophisticated*, i.e., from absolutism to multiplism to evaluativism (which were measured using a test developed by van Strien et al., 2012a).

van Strien et al. (2014) investigated the effects of prior attitudes on how students deal with conflicting information in multiple texts, indicating that students with strong prior attitudes were significantly more likely to write essays that were biased toward their prior attitudes. Moreover, students with strong attitudes took explicit stances and used large proportions of information not presented in the sources in their essays, while students with neutral attitudes wrote syntheses and used more information from the given documents.

To gain a deeper insight into the role of experience in the evaluation phase of the information search process and into the development of beliefs influencing the evaluation of information, Johnson et al. (2016) found significant differences between first-year vs. third-year undergraduates regarding the factors that influence their judgment of the trustworthiness of online information. The results indicate that the more advanced students were not only more sophisticated in evaluating information sources but also more aware in terms of making use of the evaluation criteria.

Likewise, Hsu et al. (2014) examined how students’ different levels of development of their scientific-epistemic beliefs impact their online information searching strategies and behaviors. They divided undergraduates and graduates into two groups depending on whether they employed a naïve or sophisticated strategy. They measured students’ self-perceived online searching strategies and video recorded their search behaviors. Students with higher-quality scientific epistemic beliefs showed more advanced online searching strategies and demonstrated a rather meta-cognitive search pattern.

Mason et al. (2014) studied whether *topic-specific prior knowledge and epistemological beliefs* influence visual behavior when reading verbally and graphically presented information on webpages. They found that readers made a presumably implicit evaluation of the sources they were confronted with. University students with more elaborated topic-specific epistemic beliefs spent more time on graphics in the context of more reliable sources and increased their knowledge of the topic.

The study of Kahne and Bowyer (2017) is of particular interest for our analysis, as they took policy positions into consideration, an aspect which plays an important role in the task scenario we administered to our test participants (see section “Research Questions”). In their survey of young adults, which is representative for the U.S., they asked participants to judge the veracity of simulated online postings. Controlling for political knowledge and media literacy, their main finding was that the alignment of statements with prior policy beliefs is more decisive for the evaluation of information quality than their accuracy.

Summing up, from the findings reported in recent literature, we register several commonalities in respect to the relation between *beliefs and the evaluation of internet-based information*. First, information as such and especially information encountered on the Internet was generally recognized and processed on the basis of beliefs and attitudes. Initially, students were always inclined to consider information trustworthy that corresponds with their own (prior) knowledge, whereas they tended to neglect conflicting information. Other biasing factors were prior beliefs (attitudes), which were of comparatively greater impact on the ascription of quality of information in terms of credibility, reliability, plausibility, or trustworthiness. Students appear to be liable to believe and to use information sources in line with their previous convictions, i.e., to avoid “cognitive dissonances” (Festinger, 1962). In addition, the impact of these factors is moderated by their strength (i.e., attitude strength). All in all, well-developed and more advanced (domain-specific) prior knowledge and epistemic beliefs seem to positively influence the quality of students’ Internet searches and (online) information processing.

## Research Questions

In the studies we referenced above, the question of whether (prior) beliefs and attitudes are personal traits or states and to what extent they may *change* remains open. We do not yet know whether (prior) beliefs and attitudes will change during the information acquisition process, and if so, under what circumstances. Our study aims to shed some light on the answers to these questions.

**FIGURE 1:**
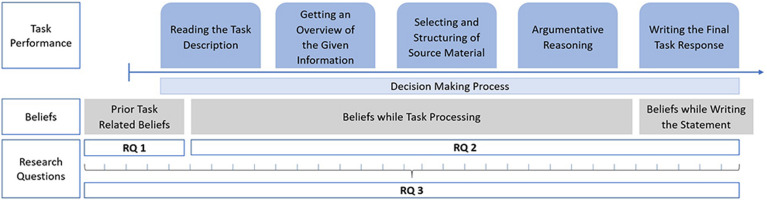
The analytical framework of the study including the research questions (*RQ*).

More specifically, based on the analyses of the current state of international research (see Section “State of Research on Beliefs and Their Impact on (Online) Information Processing”), we developed an analytical framework for our study as presented in Figure 1, and specify the following research questions (*RQs*):

(I) The Relationship between Beliefs and Decision-Making

RQ1: Students’ beliefs at the beginning of task processing

• Do the students indicate that they held certain beliefs before they began the performance assessment?
• Is it possible to identify distinct types based on these beliefs?

RQ2: The relationship between students’ beliefs and their reasoning process as well as their final decision (written task response)

• At which point in time during task processing did the students make their decision?
• Do the students’ beliefs affect their decision-making process?
• Is it possible to identify distinct profiles of decision-making?
• Which reasoning approaches become evident that may influence the decision-making of the participants?

(II) Change of Beliefs While Solving the Task.

RQ3: Interaction between students’ beliefs and the processing of the given information (in the task)

• Do the students’ beliefs change as they progress through the task and encounter multiple new information sources along the way (which could indicate that the processed information influences the students’ beliefs)? If so, to what extent is this reflected in their final decision (written task response)?

## Conceptual and Methodological Background

### Critically Reasoning From Multiple Sources of Information

Students’ skills in judging (online) information are of central importance to avoid the acquisition of erroneous domain-specific and generic knowledge (Murray and Pérez, 2014; Brooks, 2016). The abilities involved in finding, accessing, selecting, critically evaluating, and applying information from the Internet and from various media are crucial to learning in a globalized digital information society (Pellegrino, 2017; List and Alexander, 2019). Students need *critical reasoning* (CR) skills to judge the quality of the information sources and content they access inside as well as outside of higher education (Harrison and Luckett, 2019). Students need CR to recognize easily available biased and counterfactual information, withstand manipulation attempts (Wineburg and McGrew, 2017; McGrew et al., 2018), and avoid generating erroneous domain-specific and generic knowledge or arguments.^1^

In our study, we follow the definition of CR and its facets as described in Zlatkin-Troitschanskaia et al. (2019b). CR is defined as students’ *(I.) identification, evaluation, and integration of data sources; (II.) recognition and use of evidence; (III.) reasoning based on evidence, and synthesis; (IV) (causal and moral) recognition of consequences of decision-making, which ultimately lead to (V) the use of appropriate communicative action*. The performance assessments used in this study to measure CR (see next section) are based on these five theoretically driven central facets of this definition of CR. Students’ ability to critically reason from multiple sources of information as a specific representation of CR was measured within this assessment framework.

### Assessment Frameworks for Measuring Critical Reasoning

Valid measurement of CR skills is an important component of a program of research on how CR can be effectively promoted in higher education. Moreover, as part of a validity argument, CR’s relation to other related constructs needs to be examined. Based on existing psychological learning models (Mislevy, 2018; Pellegrino, 2020), analyses of this kind can provide a significant contribution to developing appropriate explanatory approaches to CR. Despite the urgency of this topic for higher education (Harrison and Luckett, 2019), theoretically sophisticated CR learning and performance assessment tools have so far been developed by only a few projects internationally (for an overview, see Zlatkin-Troitschanskaia et al., 2018a).

Multidimensional and multifaceted (meta)cognitive higher-order (procedural) skills, such as CR, can be validly measured with closed-format tests to a limited extent, as selected-response items fall at the lower end of the ‘lifelike fidelity scale.’ Multiple-choice tests predominantly assess declarative and conceptual/factual knowledge (e.g., Braun, 2019). As Liu et al. (2014) and Oser and Biedermann (2020) documented, there are several closed-format tests for assessing CR (or related constructs). One main shortcoming of tests of this kind is their limited face validity, ecological validity, or content validity (Davey et al., 2015). They usually demonstrate (extremely) strong correlations with tests focused on general intellectual ability [e.g., intelligence tests or the Scholastic Aptitude Test (SAT)], but they tend to fail to measure more specific procedural skills regarding the use and the evaluation of information sources used for learning in higher education. Well-established CR assessments have been based on standard-setting research (Facione, 1990; Facione et al., 1998), but have used multiple-choice formats and brief situational contexts and have assessed generic minimal inferencing abilities.^2^ Despite the broad use of this test type in educational assessment, it remains unclear to what extent these tasks are ecologically valid and whether students can transfer the measured abilities to more authentic and complex requirement situations.

At the other end of the assessment spectrum are traditional essay prompts with open responses and rubric scoring. Their suitability for assessing CR based on multiple sources of information in particular, may be limited by challenges in objective scoring and the brevity of the prompt (Zlatkin-Troitschanskaia et al., 2019b). While ecological validity in particular is especially limited in standardized tests (Braun, 2019), CR can be more adequately measured through *performance assessments* that simulate the complex environment students find themselves in ‘in everyday life,’ and provide an addition to standardized measures, as they are better suited to reflect current contexts and learning conditions inside and outside of higher education (Oliveri and Mislevy, 2019; Shavelson et al., 2019).

So far, to measure university students’ performance on concrete, real-world tasks and to tap their critical thinking skills, the Council for Aid to Education (CAE) has developed the Collegiate Learning Assessment (CLA) (Klein et al., 2007), which was also used in the Assessment of Higher Education Learning Outcomes study, and has launched a refined performance test on CT, the CLA+. The assessment contains an hour-long performance task and a half-hour set of multiple-choice items so as to produce reliable individual student scores (Zahner, 2013). The CLA+ is available internationally for various countries (Wolf et al., 2015). It has been used in the United States and was also adapted and used in Finland^3^, Italy, and the United Kingdom (Zahner and Ciolfi, 2018), and has undergone preliminary validation for Germany (Zlatkin-Troitschanskaia et al., 2018b). This computer-delivered assessment consists of a performance task where students are confronted with a complex scenario. Additionally, they are presented with a collection of documents with further information and data that should be used to properly evaluate the case and decide on a course of action. The test has an open-ended response format and is complemented by 25 selected-response questions on separate item stems. According to Wolf et al. (2015), the assessment measures the following student abilities: Problem-solving and analysis, writing effectiveness, writing mechanics, reasoning scientifically and quantitatively, reading critically and evaluatively, and critiquing an argument.

Other assessments that were recently developed for higher education, such as HEIghten by ETS^4^ or The Cap Critical Reasoning test, can be considered knowledge-based analytical-thinking, multiple-choice tests^5^ and do not encompass any performance tasks (for an overview, see Zlatkin-Troitschanskaia et al., 2018a).

### The iPAL Study on Performance Assessments of Higher Education Students’ CR

In iPAL (international Performance Assessment of Learning), an international consortium focuses on the development and testing of performance assessments as the next generation of student learning outcome measurements (Shavelson et al., 2019). The researchers address the question how performance assessments can enhance targeted student learning beyond rote memorization of facts and actively foster students’ acquisition of 21st century skills (including CR). The subproject presented here is designed to measure higher education students’ CR by simulating real-life decision-making and judgment situations (Shavelson et al., 2019).

The German iPAL subproject follows a criterion-sampling measurement approach to assessing students’ CR. Criterion-sampled performance assessment tasks present real-world decision-making and judgment situations that students may face in academic and professional domains as well as in public and private life. Test takers are assigned a role in an authentic holistic scenario and are given additional documents and links to Internet sources related to the topic of the task (presented in different print and online formats) to be judged in respect to their varying degrees of trustworthiness and relevance. The skillset tapped by these tasks comprises skills necessary to critically reason from multiple sources of information, i.e., to critically select and evaluate (online) sources and information, and to use them to make and justify an evidence-based conclusive decision.

In the German iPAL study, we developed a performance assessment with a case scenario (renewable energy) [comprising 22 (ir)relevant, (un)reliable and partly conflicting pieces of information]. This newly developed computer-based performance assessment was comprehensively validated according to the Standards for Educational and Psychological Testing (American Educational Research Association [AERA], 2014; see Zlatkin-Troitschanskaia et al., 2019b; Nagel et al., 2020). Validity evidence was gathered *(i)* by evaluating the test-takers’ responses to the performance assessment (for details, see Shavelson et al., 2019; Zlatkin-Troitschanskaia et al.,2019b), *(ii)* a semi-structured cognitive interview, and (*iii*) an additional questionnaire on the students’ personal characteristics such as prior knowledge, general intellectual ability, and media use behavior (for details, see Nagel et al., 2020).

In the following, we focus on the validation work conducted in *(ii)* cognitive interviews and present the analyses of transcripts of these cognitive interviews and corresponding results. To strengthen our validity argument (in the sense of Messick, 1994; Kane, 2013; Mislevy, 2018), we additionally refer to the particular findings from *(i)* to demonstrate how students’ beliefs and reasoning processes as identified in the cognitive interviews are related to their task performance (written final response on the case presented in the task).

## Materials and Methods

In this section, we first describe the entire study, including the performance task and the other assessments applied, before presenting the sub-study of the cognitive interviews and its results.

### Instruments

#### The Performance Task

To assess students’ CR and their ability to critically reason from multiple sources of information, the German iPAL study developed and tested the “Wind Turbine” performance task. This computer-based assessment consists of a realistic short-frame scenario that describes a particular situation and requests a recommendation for a decision based on information provided in an accompanying document library (including 22 snippets and sources of information of different types; e.g., Wikipedia articles, videos, public reports, official statistics). These information sources, on which the students are to base their decision recommendation, vary in their relevance to the task topic and in the trustworthiness of their contents (for detailed descriptions of the performance task, see Shavelson et al., 2019; Zlatkin-Troitschanskaia et al.,2019b).

In this case scenario, test takers were assigned the role of a member of the municipal council of a small town confronted with the opportunity to build a wind farm on its grounds. They were asked to review the information sources provided in the task and, based on the evidence, to write a policy recommendation for a course of action, i.e., to recommend to the city council whether or not to permit the construction of the wind turbines in its agricultural countryside (for more details, see Shavelson et al., 2019; Zlatkin-Troitschanskaia et al., 2019b).

#### Accompanying Assessments

To control for task- and topic-related prior knowledge, we used a short version of the WiWiKom test, which was comprehensively validated in the representative nation-wide WiWiKom study as an indicator of knowledge in economics and social sciences (Zlatkin-Troitschanskaia et al., 2019a). As two indicators of general cognitive ability, the scale “Choosing figures” of the Intelligence Structure Test (IST-2000 R, Liepmann et al., 2007) as well as the grade of university entrance qualification were used in the present study (for details, see Nagel et al., 2020). The participants’ levels of interest in the task topic and case scenario (renewable energy) as well as their test motivation were also assessed in this study using two five-point-Likert-type scales (validated in the previous studies cited).

Furthermore, socio-demographic information and personal characteristics (e.g., scales on ‘media use,’ ‘need of evaluation,’ ‘information overload’; for details, see Nagel et al., 2020) expected to affect test performance were collected. Indicators of relevant expertise in the context of solving the performance task, such as completed commercial or vocational training, were also surveyed, as they might also influence task performance.

### Study Design and Validation

To test and validate the “Wind Turbine” task in accordance with the Standards of Educational and Psychological Testing (American Educational Research Association [AERA], 2014), assessments were conducted with a total of 95 undergraduate and graduate students from different study domains (e.g., business and economics, teacher education, psychology) at a German university (Shavelson et al., 2019; Zlatkin-Troitschanskaia et al., 2019b).

The students worked on the task in a controlled laboratory on computers configured specifically for this assessment and had no access to other resources to solve the task. The study was carried out in small groups on several dates under the supervision of experienced test administrators.

The total test time for the performance task was 60 min. The time limit put the participants under pressure, which led to them not having enough time to study all given sources intensively. Instead, it required them to decide which sources and contents to select and review more thoroughly, considering their relevance, validity, and trustworthiness. After the performance task (and a short break), the participants were asked to work on the accompanying assessments. The participants received an incentive of €20, and were offered an individual feedback on their test results.

Test performance was scored using a 6-point Likert-type anchored rating scheme based on the CR definition with 5 dimensions and 23 subdimensions (Section “Sample and Data”; for details on scoring, see Zlatkin-Troitschanskaia et al., 2019b). The individual task responses, i.e., short essays, were randomly assigned to two of four trained raters, and the written responses were evaluated according to this newly developed and validated scoring scheme with behavioral anchors for each sub-category. Two raters independently evaluated the participants’ task responses, and a sufficient interrater agreement was determined (Cohen’s κ > 0.80; *p* = 0.000, for the overall test score).

In terms of psychometric diagnostics, the student response when solving the performance task (well-founded written final decision) was interpreted as a manifestation of their latent (meta)cognitions. The students’ task performance, i.e., their written responses, were regarded as valid indicators of the students’ ability to critically reason from multiple and contradictory sources of information (in the sense of the construct definition, see Section “Critically Reasoning from Multiple Sources of Information”). The theoretically hypothesized multidimensional internal structure of this construct was supported empirically using confirmatory factor analysis (CFA) (Zlatkin-Troitschanskaia et al., 2019b).

As theoretically expected, analyses of the task performance did not identify any significant domain-specific effects among students from different study domains (Nagel et al., 2020). This also holds true for prior knowledge from previous vocational training, which showed no significant effect on the test results. As the performance task was developed to measure generic CR skills, this finding indicates that as expected, the assessment is not domain-specific. However, with regard to theories in learning and expertise research, it could be assumed that domain-specific expertise, though acquired within a certain domain, can be transferred to generic problems or tasks (Alexander, 2004). In this respect, these results indicate that students may have deficits in their (meta)cognitive abilities that would enable them to transfer their prior knowledge and skills to the new context encountered in the performance task. Overall, the results from these validation studies provide evidence of the technical quality of the developed performance task and provided evidence as to the test’s construct validity and reliability (Zlatkin-Troitschanskaia et al., 2019b; Nagel et al., 2020).

### Cognitive Interviews and Qualitative Analyses

The analyses of test performance *per se* do not permit us to draw valid conclusions on students’ underlying response processes when performing this task. In accordance with our construct and test definition, we expect that while working on the performance task, the test participants selected and evaluated the given information with the goal of finding relevant and reliable/trustworthy information for their evidence-based reasoning, decision-making, and final conclusion in the written response. To investigate how the test participants dealt with multiple contradictory sources, to what extent they integrated and evaluated given information during their reasoning and decision-making process, and which individual factors influenced their response processes, a semi-structured cognitive interview with stimulated recalls was conducted immediately after the performance assessment with a subsample of participants (see next section). The participants were first shown a screen displaying all 22 documents included in the document library one after the other. The students reflected and commented on, for instance, whether they considered the source in question and the information given therein to be relevant and/or credible (and why), and whether they used or ignored this source and information (and why). Particular attention was paid to controlling whether the students were aware of their task topic-related beliefs and attitudes and if so, whether they were aware to which extent these influenced their critical reasoning while processing the task, for example resulting in selective inclusion of the given information. The interviews took approximately 80 minutes and were recorded for later transcription.

The interview questions included, for instance, whether a test participant held task topic-related beliefs about wind power, the environment, etc. prior to the performance assessment, and to what extent previous experience, individual knowledge, attitudes and beliefs relevant to the task topic influenced the students’ information selection, evaluation and decision-making. In particular, the students reported at which point in time during the task processing they made their decision whether or not to suggest to the municipal council to allow the building of the wind farm (for instance, indicating that many students made their decision before they had even read the given information; see Section “Results”). The cognitive interviews also indicated that the performance task with its task prompt is clear and suitable for the objectives of the presented study.

The evaluation of the data from the cognitive interviews in the German iPAL project was carried out using the software MaxQDA. Based on the cognitive interview protocols, a differentiated category system was developed and validated. More specifically, the qualitative analysis of the cognitive interviews was guided by grounded- and data-driven theory for developing a coding scheme (Strauss and Corbin, 1998).

The iterative process of coding consists of (1) open, (2) axial (Strauss and Corbin, 1990) and (3) selective coding. At first, an open coding was used to access the data that did not yet follow any schematics. In the subsequent step, first categories were identified, such as the students’ beliefs at the beginning of the task or at which time point during the task processing the decision was made. Then, all interviews were analyzed and coded based on the defined categories. The coding scheme highlighted the points of reference regarding different information sources used by the students in their interviews. It linked the use of different sources to the students’ reasoning processes while reaching their decision, making it possible to derive and generalize response patterns. Coding development was complemented by an analytical approach of constantly comparing phenomena within the dataset (minimum and maximum contrast between the phenomena). Selected codes with a focus on student beliefs are presented in this paper (see Section “Results”).

The classification of the participants into the three profiles described in Section “Results” was based on a combination of criteria from the category system. These were primarily: (1) at what point during the assessment they made their decision (reported in the cognitive interviews), (2) their decision-making process (pros and cons; intuitively, based on original opinion; based on a specific source, etc.) and (3) the strength of their beliefs (strong personal beliefs primarily about nature conservation/animal welfare, etc. and general personal identification with the task topic). As the participants were classified into profiles based on a combination of these three categories, participants classified into different profiles may share certain attributes (e.g., listing pros and cons).

### Sample and Data

The semi-structured interviews were conducted with a purposefully selected subsample^6^ (which is part of the overall sample used in the German iPAL study) of 30 university students from one German university and from different courses of study. With this subsample, we aimed to include students from all study domains represented in the main sample in the cognitive interviews as well. Accordingly, the subsample consists of about 50% students of economics education, while the other half comprises students from other study domains (economics, psychology, and geoscience). Another important criterion for purposeful sampling was to include as wide a range as possible in terms of participants’ prior study experience and other personal characteristics that may influence students’ task topic-related beliefs, attitudes and knowledge, and their task performance. Accordingly, the subsample consists of students from both undergraduate and graduate programs and in different study semesters. To gain first indications of the possible impact of knowledge and skills achieved during academic studies, we focused on more experienced students. The average duration of studies to date among subsample participants was therefore 5.1 semesters, indicating that the students were fairly advanced in their respective study programs. Additionally, the university entrance qualification (with an average grade of 2.1; range from 1.0 = best to 6.0 = worst; *n* = 25^∗7^. To control for the possible impact of pre-university education and practical experience, we included students with completed vocational education and training (11 students had completed an apprenticeship before beginning their academic studies; *n* = 29^∗^^7^). The average interviewee’s age was 24 years; 21 students were female – these proportions were similar to those in the overall sample.

Despite this purposeful sampling procedure, as participation in this study was voluntary, our sample cannot be considered representative. However, no significant deviations from the entire student population described in Nagel et al. (2020) were found with regard to the socio-biographical characteristics (e.g., gender and age).

## Results

### Prior Findings on Test Performance and Additional Assessments

The students achieved an average intelligence-test (IST) score of 17.18 points (out of a maximum score of 40 points; *n* = 28^∗^^7^) and an average economics knowledge test score of 10.46 points (out of a maximum score of 15 points; *n* = 23^∗^^7^). Only four students stated that they had previous practical experience with Wind Turbines. Most students reported a low to medium level of task topic-related previous knowledge while a high level of knowledge on wind turbines was very rare (*n* = 1).

The mean performance on the task was 3.52 points with 6 points being the highest possible score (for the scoring, see Zlatkin-Troitschanskaia et al., 2019b). The median number of information sources students used in their written statements was 7 (out of 22 information sources given in the task). The written argument-based statements within the scope of the task processing differed in length, which was on average 426 words, with a maximum of 866 words and a minimum of 68 words, indicating a high deviation (*SD* = 196 words) within the sample.

In the following, these results were used as external criteria to demonstrate how the following results from the analyses of the cognitive interviews correspond to these results of the quantitative analyses of the test scores.

### The Relationship Between Beliefs and Decision-Making

#### RQ1: Students’ Beliefs at the Beginning of Task Processing

In the cognitive interviews, the students were asked whether they had been aware of their task topic-related beliefs prior to working on the task and if so, whether they were aware that their personal beliefs may have influenced their decision in the performance task and how they believed this influence may have shaped their response. Most participants (*n* = 23) stated that *they had already held certain beliefs on the task topic before beginning the task*. For instance, one participant stated: “[…] *I think I would have recommended this from the beginning because this is also a topic I hear about in the media from time to time, so that I already have a personal opinion about wind power and energy*“(ID15). In response to the question whether his personal beliefs had influenced his response, interviewee ID7 stated: *“Sure, because then I did not even look at the controversial sources at all and, that is… for example, if I believed that the bats from source 21 were extremely important, then of course I would have looked at the source.”* Seven participants (*n* = 7) reported that they did not have any prior beliefs about the topic of the performance assessment.

In terms of distinct types based on the reported beliefs, both groups of students – those who indicated prior beliefs and those who did not – can be further distinguished into two subgroups each *(i)* depending on the students’ *positive or negative stance toward wind turbines*, which vary considerably in stance strength and *(ii)* which can also be linked to a *more economics-focused or a more ecologically oriented reasoning perspective* (see Section “The Relationship between Beliefs and Decision-Making”).

#### R2: The Relationship Between Students’ Beliefs, Their Decision-Making Process and Their Final Decision

##### Time of the decision-making and types of decision (intuition-based vs. evidence-based)

In the cognitive interviews, the students reported *at which point in time while working on the performance task and processing the information they made their decision* as to whether renewable energies should be promoted or not in the given case (see Table 2). About one third of the students (*n* = 8) made their decision *at the beginning of the task, after having read the scenario, even though they had not yet read or considered the given information* at all or only very briefly.

**TABLE 2:** Time of decision-making and type of decision.

Time of the decision-making	Frequency	Average test score
Early, intuitive decision (before source evaluation)	8	3.76
Decision after reading (selected) sources (multiple) times)	8	3.11
Late, intuitive decision (after source evaluation)	5	3.25
Late decision based on pro-/con- arguments	9	3.83

Another group of students (*n* = 8) used the given sources and made their decision mostly after (more or less thoroughly) looking through the information provided. For instance, when asked when he had decided in favor of or against the construction of wind turbines, interviewee (ID 1) stated “*yes […], I actually knew from the beginning when I went through this [task] what direction my statement would go in.*” Interviewee (ID 23) made his decision while working on the audiovisual information: “*So after I watched the videos […] I changed my opinion*.”

In contrast, other students made their recommendations after having reviewed the information material *and after weighing up the pros and cons* (*n* = 9), as it is the case with, for example, interviewee (ID 7): “Interviewer: *So that means that you first read all the sources and all the arguments?* Participant: *First the pros and cons, and only then I had a feeling*.” This finding indicates that for some students the creation of pro and contra lists was an important step in their decision-making process. However, not all of those who made their decision comparably late in the task-solving process stated that they had done so based on weighing up the pros and cons: five students indicated that they made a late but still *intuitive decision*.

Overall, with regard *to the time of decision-making*, four types can be distinguished among the participants (Table 2), which differ in terms of *intuition-based vs. evidence-based* decision-making as well as the extent to which the given information and pros/cons were considered or ignored.

Students who made a late intuition-based decision (*n* = 5) performed worse, with an average test score of 3.25. Students who made their decision at the end of the task based on weighing pros and cons (*n* = 9) performed better compared to all other participants, with an average test score of 3.83. Noticeably, there were hardly any differences in task performance between the students who decided intuitively at the beginning of the task and the students who weighed up pros and cons and decided at the end of the task.

#### The Relationship Between Students’ Beliefs and the Decision-Making Process: Profiles of Decision-Making

Regarding the question to what extent students’ were aware that their beliefs impact their decision-making process and whether distinct profiles of decision-making can be determined, among the 30 study participants, we identified students who indicated that their previous beliefs played a *major*, *minor* or *no role* in their decision-making process. Combined with the time at which they made their decision, we distinguished three profiles of decision-making:

*Profile 1 “determined”*: Participants who ignored the given information and made their decision solely based on their individual beliefs, almost immediately after having read the task (*n* = 7). For example, (ID5) stated: *“I wouldn’t have made a recommendation that goes against my gut instinct. For example, I think that even if the sources had been chosen in such a way that they would have given me a negative impression, I am not sure whether that would have caused me to change my initial positive stance. I simply couldn’t just ignore my background knowledge and my personal attitude when giving my recommendation at the end.”*

*Profile 2 “deliberative”*: Participants who decided contrary to their task topic-related beliefs, and changed their decision after having read the information provided in the task (*n* = 11), as well as participants who stated that they held certain beliefs at the beginning of the task but weighed up pros and cons while processing the task and made their decision based on these considerations (*n* = 5). The two cases were merged into one profile as students in both cases stated that they held certain beliefs but made their decision based on the pros and cons of the evidence rather than on those beliefs.

There were some differences within this decision-making profile. For instance, some students switched between being in favor of or against the construction of wind turbines while working on the task: “*So basically I’m for it and then while I was writing this I just started to waver, you have to list the negative things and then I doubted it for a moment but then I finally decided in favor at the end.*” (ID 8); other students changed their prior opinions by reflecting on their own beliefs in the context of the given information: “*At the beginning I would have said yes [impact of belief on decision-making]. But then I tried to be as unbiased as possible, or rather to be subjective in my role as a member of the council. And then I kind of abandoned my [initial] decision and my personal belief.*” (ID 17).

*Profile 3 “open minded”*: Participants who did not state any prior beliefs, took note of the provided information, and made their decision after considering pros and cons (*n* = 7). Interviewee (ID7) stated that he had had no prior beliefs before starting the task, and that he made his decision after considering the given information and making a pro and con list: “*No, I couldn’t decide at the beginning, it just happened toward the end of the argumentation. Well, I was not for or against it from the beginning. I just did not know how to decide.*”

Since the participants were classified into profiles based on a combination of the scoring categories (see Section “Cognitive Interviews and Qualitative Analyses”), participants classified into different profiles may share some attributes (e.g., listing pros and cons) and there may be some overlaps between the profiles.

#### The Relationship Between the Decision-Making Profiles and Task Performance (Test Scores) as Well as the Results of Additional Assessments

Noticeably, the participants in profile 3 on average achieved higher test scores than the other two profiles (Table 3). Students who based their decision on their beliefs (profile 1) performed worse compared to other participants (profile 3). In terms of the average test score, the deviation between these two profiles (1 and 3) was more than 1 point.

**TABLE 3:** Means of task performance of different profiles.

Impact of students’ beliefs	Frequency	Average test score	*SD*
**Profile 1 “determined”:** Decision based on firm beliefs prior to processing the task	7	2.95	1.10
**Profile 2 “deliberative”:** Decision made after reflection on beliefs	16	3.38	0.59
**Profile 3 “open minded”:** Decision made after a neutral approach, primarily reflecting on the source material	7	4.01	0.44

Upon further characterizing the three profiles, we found additional differences between the groups of students in terms of the number of information sources used and the number of words in the final recommendation statements, which differ greatly (Table 4). Compared to students who made a decision based on their beliefs (profile 1), the average number of information sources used was 3.25 points higher for students who changed their beliefs (profile 2) and 2.86 points higher for students of profile 3. The mean number of words in the written final recommendation statements also varied heavily. Remarkably, the responses of “deliberative” students (profile 2) were the shortest with an average of 370 words. “Determined” students (profile 1) who did not change their beliefs wrote on average 33 more words than “open minded” students (profile 3) with a mean of 473 words.

**TABLE 4:** Characteristics of the three decision-making profiles.

Profiles	Profile 1 “determined” (*n* = 7)	Profile 2 “deliberative” (*n* = 16)	Profile 3 “open minded” (*n* = 7)	Total Sample (*n* = 30)
Number of Information Sources Used	5	8.25	7.85	7.5
Length of Written Response	506	370	473	426
Gender	Female: 7	Female: 8	Female: 6	Female: 21
	Male: 0	Male: 8	Male: 1	Male: 9
Age (*n* = 29)	21.17	24.75	24.43	23.93
Degree	Bachelor: 6	Bachelor: 14	Bachelor: 6	Bachelor: 26
	Master: 1	Master: 2	Master: 1	Master: 4
Vocational Training (*n* = 29)	Yes: 2	Yes: 5	Yes: 4	Yes: 11
	No: 4	No: 11	No: 3	No: 18
Intelligence Test Score (*n* = 28)	16.67	17.40	17.14	17.18
Economic Knowledge Test Score (*n* = 23)	10.20	10.65	10.20	10.35
University Entry Qualification Grade (*n* = 29)	1.87	2.18	2.17	2.11

With regard to personal characteristics, there were no significant differences in the intelligence test scores for the three profiles (Table 4). The same was true for performance in the economics knowledge test, with results ranging from 10.20 to 10.65 points (on a 15-point scale).

Students who made their decision based on evidence and pros/cons, despite their beliefs or without considering previous task-related beliefs, tended to be older (profile 2: 3.58 years older on average; profile 3: 3.26 years older on average) than “determined” (profile 1) students. There were no significant differences in terms of gender, pre-university education (vocational training or university entry qualification grade) or degree course, which does not indicate any substantial influence of prior education on the response processes.

#### Task-Topic Related Attitudes and Their Relationship to Reasoning Processes and Decision-Making

Another approach to identifying certain beliefs and their possible relationship to information processing and critical reasoning was to analyze students’ task-topic related attitudes and their impact on reasoning approaches when solving the performance task. In this respect, the reasoning lines identified in the cognitive interviews (as well as in the written task responses) can be categorized as follows:

(1) The first category differentiates between primarily *economics-focused* or *ecologically oriented* reasoning lines. Twelve students’ recommendations had a primary economical focus in their reasoning, while 18 students relied more on ecological aspects and sources presenting an ecological perspective.

Remarkably, students who were in favor of building wind turbines tended to choose an economics-focused reasoning line, while students against the construction chose an ecologically focused perspective (Table 5). An example for an economical reasoning line can be seen in the following statements: “*The trade tax to be paid by the operator could be sensibly invested in the modernization of facilities, the infrastructure of the place and the marketing of the local recreation area. This source of income seems to be important for the community, especially in the future, against the background of an increasingly dwindling agriculture” (ID 13); “In my opinion, the offer should be accepted, as the positive aspects outweigh the negative ones and, in general, the construction of wind turbines would mean a macroeconomic, long-term benefit for the community. In addition, it is an investment in infrastructure.”* (ID 25). In contrast, an example for an ecological line of reasoning and their relationship to information processing and decision-making can be seen in the following statement (ID 26): “*That caused me to have fewer choices, and I had already had the notion in mind that wind turbines are good and nuclear power plants are bad, which is why I said from the very outset that yes, no matter in which form, more renewable energy should be produced and, well, that’s why I said all along that that would be the most sensible result in my opinion, without any of those arguments.”*

**TABLE 5:** Economic- and ecological-focused reasoning and decision against or in favor of wind turbines at the end of the tasks.

Reasoning Approach Frequency/(Average test score) *n = 29*	In favor of building wind turbines at the end of the task	Against building wind turbines at the end of the task
Economic-focused reasoning	9 (3.68)	3 (3.82)
Ecological-focused reasoning	6 (3.95)	11 (3.17)

(2) The students’ decision-making processes and (final) recommendations can also be categorized in terms of the extent *to which the specific situation described in the task was considered*. While half of the participants took the *task-specific perspective* of the local council and the current situation of the city into account (*n* = 15), other students choose a more general approach in making a recommendation for or against wind turbines (*n* = 15).

One example for considering of local conditions can be found in the statement of participant (ID 7): “*I consider the construction of the wind turbines in the north of the municipality to be an incalculable risk, as the tertiary sector and especially the tourism that goes along with it represent an important source of income for the town. I think it makes much more sense to locate the wind turbines in the west. Farmers who live there, such as Mr. Anders and Mr. Bender, should welcome an additional source of income besides agriculture, so that they should agree to the construction of the wind turbines.“*A more general approach is expressed in the statement of participant (ID 16): *“The fact that wind energy is initially a clean and environmentally friendly way of generating energy speaks for the installation of wind turbines. In addition, there are also economic reasons for this, as good money can be made from the rent that incurs when a wind turbine is installed. […].”*

While the majority of students who took the task-specific current situation of the city into account tended to express a negative attitude about wind turbines, students who took a more general reasoning approach were rather in favor of building wind turbines (Table 6).

**TABLE 6:** Perspective of reasoning (local council included or not) and decision against or in favor of wind turbines at the end of the tasks.

Reasoning Approach Frequency/(Average test score) *n = 29*	In favor of building wind turbines at the end of the task	Against building wind turbines at the end of the task
Included perspective of local council in the decision regarding the construction of wind turbines	5 (3.97)	10 (3.37)
Made a general decision regarding the construction of wind turbines	10 (3.70)	4 (3.16)

#### The Relationship Between the Reasoning Approaches and Task Performance (Test Scores)

In terms of task performance, no significant differences were found between the students with different reasoning approaches, although students who chose economics-focused reasoning achieved slightly higher performances than the other students. When taking into account the positive vs. negative stance toward wind turbines at the end of the task, however, the difference in task performance of students with ecological-focused reasoning is about 0.8 points, whereas the difference in the group with economic-focused approach is only 0.1 point.

### Change of Beliefs While Solving the Task

#### RQ3: Interaction Between Students’ Beliefs and Processing of the Given Information

Looking at the time of decision-making, we found that some students changed their opinion about the construction of wind turbines (once or several times) while processing and working on the task, while others did not. While 14 interviewees reported that they did not change their opinion about the wind turbines over the course of their task solving, 12 interviewees changed their opinion after processing of information given in the task (Table 7). Four participants claimed that they had not been initially disposed either way. Both groups of students–those who changed their opinion and those who did not–can each be further distinguished into two subgroups depending on their positive or negative stance toward wind turbines, which vary considerably in size. Within the group with no change of opinion, participants who had voted against the construction on wind power plants at the beginning of the tasks and remained negative (*n* = 3) can be distinguished from participants who had a positive stance toward wind turbines before and after completing the task (*n* = 12). We can also differentiate between students who have changed their opinion during working on the task. Some students initially had negative attitudes toward wind turbines, but changed their opinion during the task processing and in the end voted in favor wind turbines (*n* = 2). The same applies to participants who were in favor of constructing wind turbines at the beginning, but ultimately spoke out against wind turbines (*n* = 9).

**TABLE 7:** Change of opinion.

Change of opinion about wind turbines:	Frequency	Average test score
Change of opinion	12	3.17
No change of opinion	14	3.86
(No statement at the beginning and/or end)	4	3.24
**Subdimensions: Change of opinion about wind turbines: Student…**		
…maintained a positive stance towards the construction of wind turbines	12	3.83
…maintained a negative stance towards the construction of wind turbines	3	3.96
…changed from positive to negative stance towards the construction of wind turbines	9	3.12
…changed from negative to positive stance towards the construction of wind turbines	2	3.39

#### The Relationship Between a Change of Students’ Beliefs and Task Performance (Test Scores)

There was hardly any significant difference in the test score of the two groups, although students who did not change their opinion performed slightly better than students who changed their opinion: the difference in task performance was about 0.7 points.

## Discussion and Conclusion

### Summary and Interpretation of Results

The data from the cognitive interviews on the students’ beliefs, information processing and reasoning processes make a valuable contribution to explaining the students’ CR abilities and the complex interplay between their underlying thought processes and task topic-related beliefs. In the interviews, most participants expressed that they were aware of holding certain beliefs at the beginning of task processing *(RQ1).* The results of the qualitative analysis of the cognitive interview protocols indicated that the students’ task topic-related beliefs had an influence on their selection, critical evaluation and use of information as well as on their reasoning process and final decision *(RQ2).* As an additional decisive contribution to existing research [see Section “State of Research on Beliefs and Their Impact on (Online) Information Processing”], we provide initial evidence that some students’ task topic-related beliefs changed over the course of task processing, indicating that the processed information (recognized and reflected evidence and pros/cons) influenced the students’ beliefs to varying degrees *(RQ3).*

Overall, the evidence from this qualitative analysis suggests a complex reciprocal and changeable relationship between students’ task topic-related beliefs, their processing of new (confirm or deviant) information and their decision-making based on both beliefs and evidence.

More specifically, the types of beliefs and attitudes derived from the cognitive interview data suggest their influence on information processing, reasoning approaches and decision-making. In particular, the students who already had strong task topic-related beliefs at the beginning regarded these as decisive while solving the task. For instance, students who had already made a decision based on their beliefs at the beginning of the task cited fewer sources in their written response (final decision).

Overall, the *selection, evaluation, and use of information* while working on the task were influenced, in particular, by the participants’ *task topic-related beliefs (RQ2)*. By contrast, hardly any differences became evident in terms of students’ relevant knowledge. However, the majority of the participants had only little prior knowledge of the subject, i.e., a large amount of the information in the task was new to them. Though most students had a positive or negative stance toward renewable energy in general, their personal beliefs concerning wind energy in particular did not appear to be very firm and well-founded. The few test participants who had already dealt with the subject area in more detail appeared to have more solid personal beliefs about wind energy *(RQ1).* Furthermore, there were no differences in terms of students’ general interest in the topic. However, two reasoning lines – more ecologically oriented vs. economics-focused approaches – became evident, which appear to influence students’ decision-making processes and final decision.

Remarkably, the students who had more elaborated beliefs prior to processing the task were more likely to come to a decision that contradicted their personal beliefs. For instance, the information on the negative effects of wind turbines on the health of people and animals living in the vicinity of a wind farm (noise emission, bird strike, infrasound) was particularly relevant for these participants when making their decision; they were more astonished by this information than the students who had hardly any prior knowledge about the subject and no well-developed beliefs *(RQ2).*

Most students started selecting information right away after obtaining an initial overview of the sources presented in the task. The participants’ subsequent evaluation of the given information with regard to the *reliability, validity, objectivity, and trustworthiness* of the respective sources (as stated in the interviews) does not appear to have had much of an influence on their selection and use of information. In contrast, the participants evaluated the *relevance* of the sources differently, whereby a large number of the sources that were evaluated as relevant were used to inform their decisions and help them formulate their written recommendations. For instance, in the interviews, the majority of students rated Wikipedia as a less reliable source (of course the exact details vary, but in general, it received rather negative ratings), as Wikipedia pages can potentially be edited by any Internet user. However, the choice as to whether or not to use information from Wikipedia sources was primarily made on the basis of the *content* of these sources (“*do I want to address bird mortality or not?*”). In contrast, when it came to the evaluation of the public-service broadcaster videos, a large number of participants assessed these videos as trustworthy despite not having watched them, as they considered this source to be particularly reliable.

Overall, in the cognitive interviews it became evident that the students mostly selected and evaluated (or ignored) new information depending on media or source type (i.e., whether they believed that certain types of media and presented sources are relevant and reliable) but not on the particular content/evidence. This finding is in line with previous research reported in the Section “State of Research on Beliefs and Their Impact on (Online) Information Processing” and stresses the importance of epistemic beliefs regarding information sources, which was not a focus of this study and requires further investigations in the particular context of online reasoning (for limitations, see the next section). In addition, this result points to a demand for more observational studies that capture in detail what documents, what parts of these documents, and which content the participants read and comprehend while solving the task.

Although participants used different *sources* in their statements, most of the students did not compile the information provided to them and weigh the evidence (pros/cons), but rather selected information related to their own beliefs, indicating biased selection, evaluation and use of information (for the confirmation bias, see Mercier and Sperber, 2009, 2011; Metzger et al., 2010; Metzger and Flanagin, 2015). A (repeated) critical examination of the information and evidence provided did not take place.

Linking the results from the qualitative analyses of the cognitive interviews with task performance further suggests a confirmation bias in reasoning, showing that students who only made their decision based on their beliefs (profile 1) had the worst test scores on average. This was also reflected in the number of sources used. They wrote the longest statements but based on the lowest number of used *sources*, without sufficiently reflecting on the available information and evidence. This finding is also supported by the lower performance of students who tended to overemphasize a single source while neglecting all contradicting source information (for the authority bias, see Metzger et al., 2010; Metzger and Flanagin, 2015). Overall, the finding from the qualitative analyses that often no sufficient critical reasoning took place in the decision-making process and that the decision was based on beliefs (and bias) was also reflected in the students’ statements.

In contrast, the students with no early inclination (profile 3) approached more source material neutrally and decided on the incorporation of the information and evidence individually, outperforming the other students in terms of task score. Their statements were less belief-driven since they addressed the specific task scenario and prioritized the town’s needs and restrictions over their personal stance on renewable energy.

As the students only had limited time (60 min) to respond, time pressure also played an important role and forced them to gather relevant information as quickly as possible. If the participants selected the information they intended to read more precisely, worked with it and then used it in their decision-making at an early stage (*right at the beginning*), quickly (*without deliberative thinking*), and consistently (*without changing their minds*), the issue of time pressure apparently did not have much of an effect on their task-solving efforts. The cognitive interviews indicate that for some students, however, selecting suitable information was a major challenge while working on the task (indicating the higher cognitive load; Sweller, 1988). These participants often opted to use internal sources as opposed to external sources, indicating that they mostly focused on the information that was available within the task document itself and disregarded the hyperlinks. The majority of participants did not watch the two videos (completely) due to time issues. This aspect also points to some limitations of our study (see next section).

### Limitations and Implication for Future Research

Though the study provides some important insights into the complex reciprocal relationship between students’ beliefs and their reasoning and decision-making process, some limitations (besides those related to the sample, see Section “Sample and Data”) must be critically noted, which indicate some perspectives for further research.

While the results of the qualitative analyses pertaining to *RQs* 1&2 allow for some clear statements about students’ beliefs and their influence on critical reasoning, the findings pertaining to *RQ*3 regarding changes in beliefs are still limited. First, in our study, we can only derive conclusions about task topic-related beliefs. These need to be distinguished from general personal (e.g., epistemic) beliefs, which were not analyzed in our study. In prior research, general beliefs usually were seen as a trait that does not change during the course of solving a task. However, measuring epistemic beliefs is considered challenging from a conceptual as well as a methodological perspective, and requires further research (van Strien et al., 2012a).

Second, based on the cognitive interview protocols, a clear distinction between a change in task topic-related beliefs and a change of overall opinion could also only be made to a limited extent. Although some students clearly stated that they had beliefs prior to processing the task that influenced their information processing and decision-making, and they had changed their opinion, we cannot conclude, on the basis of the interviews, whether this *change of opinion was due to a change in their underlying beliefs*. It is also questionable whether students were able to clearly distinguish between their belief, their attitude toward the task topic, and their opinion, and to express this difference in the interviews. This limitation results in an important follow-up for further research: Is a change of opinion accompanied by a change of task topic-related beliefs?

Though the results of both assessed scales on students’ interest in the task topic and students’ test motivation showed (very) high levels among all participants in this study, we noticed some differences in the way students approached the cognitive interviews. While some students were very communicative and talked a lot about their beliefs and task processing, other students gave short answers. Consequently, the cognitive interview protocols vary substantially in length and detail. The results of the qualitative analyses must therefore be viewed critically in terms of this data limitation. For instance, it could not be ruled out that students who did not express that they had topic-related beliefs prior to processing the task may not have deliberatively reflected on this interview question or simply not have wanted to share this information (e.g., due to a bias of social desirability). Despite the use of a standardized guide in the semi-structured interviews, the comparability of the cognitive interview protocols may be limited in this regard.

The task topic may also be not without bias in this respect, since renewable energy can be generally framed in a positive light. For this reason, it can also not be ruled out that students’ responses to the task and their answers to the interview questions were biased in terms of social desirability. However, the fact that some students in our sample were both initially and ultimately against the construction of the wind turbines (*n* = 3) may contradict this assumption.

In addition, though (i) the task prompt to write an evidence-based statement regarding the decision for the community should have been clear and strong enough to indicate that a discussion of the evidence (pros-cons) made available in the task was required, and (ii) (very) high levels of assessed interest in the task topic and overall test motivation among participants were determined, a difference among participants in terms of (metacognitively) engaging their critical reasoning skills when solving the performance task can still not be ruled out. Based on prior research, however, it can be assumed that the activation of critical reasoning abilities requires metacognitive skills (e.g., Brand-Gruvel and Stadtler, 2011). Therefore, further understanding of students’ (metacognitive) engaging (and other influences) during the decision-making process is required to help identify certain patterns in task processing strategies for this type of performance assessment and to further improve computer-based simulations in terms of their ecological validity and reliability to ensure more authentic assessment (for a critical discussion, see also Mercier and Sperber, 2011).

In this context, it is remarkable that the group of students who were aware of the influence of their beliefs – despite the task prompt asking them to include the given information and evidence in their decision-making process-decided to use only information that supported their beliefs (profile 1). These students had already recognized at the beginning of the task processing that their beliefs would have a decisive influence on their decision. If we transfer this finding to other real-life situations, in particular the everyday use of online sources in Internet searching, further research is required as to whether students, when searching for sources and in the context of their university education, also specifically focus on sources and information that confirm their beliefs. In this respect, the identified reasoning profile 1 may lead to an acquisition of biased (domain-specific) knowledge. In contrast, the “open minded” profile 3 approached more information neutrally, outperforming the other students in terms of the scored quality of written statements.

In this context, it is also important to focus on those students who claimed to have certain beliefs on the topic before starting the task but still reviewed all the information given and even partly decided against their beliefs after having regarded all information (“deliberative” profile 2). This profile should be analyzed more in-depth, especially taking into consideration both additional underlying cognitive and non-cognitive student characteristics as well as specific learning opportunities that this group might have had to develop this deliberative reasoning approach. Here, further questions arise: Why did students choose this approach and decide against their beliefs? What personal or contextual factors may have played a decisive role?

The complex relationship between prior knowledge and beliefs also requires further in-depth investigation. Ho et al. (2008) found that task topic-related beliefs interact with the amount and quality of topic-relevant knowledge, whereby the topic-related beliefs may have a stronger impact on decision-making than knowledge. Analogously, the results of our study suggest that in general, no matter how experienced a student is in a topic or how much previous knowledge they had, certain beliefs seemed to be influential and predominant. However, to what extent the beliefs influence students in their approach to a task topic and which aspects were particularly crucial for students to be influenced by their beliefs (e.g., strength of beliefs or additional personal characteristics) must also be analyzed in further research (for an overview, see Brand-Gruvel and Stadtler, 2011).

In addition, looking at the differences in the students’ reported reasoning processes, we can conclude that diverse students’ beliefs and attitudes, which were related to the task context and topic to a very different extent (e.g., in the area of sustainability), had an influence on the students’ decision-making and final decision. Based on the data from the cognitive interview protocols, however, we were not able to analyze the complex relationship between beliefs, reasoning approaches and *lines of argumentation*. Though critical reasoning is indeed related to aspects of argumentative skills, this latter aspect was not the focus of our study (as described in Section “Conceptual and Methodological Background”) and requires further investigation in several regards. Particular investigation of argumentative skills would require a substantial change and further development of the experimental and assessment setting. For instance, there are several performance tests available that specifically focus on measuring argumentative skills (e.g., Argument Structure Test, Münchow et al., 2020; Agrument Judgment Test, Münchow et al., 2019) and are suitable for discriminant validation of CR assessments, which should be investigated in a follow-up research. In addition, comprehensive qualitative analyses of both the argumentative importance of the material on the one hand as well as (i) arguments (more or less reflective or intuitive, Mercier and Sperber, 2011) used by students in their responses and (ii) (new) arguments created by the students themselves based on given arguments in the provided information on the other hand need to be conducted in further studies, and explicitly linked to students’ critical reasoning ability and performance.

Finally, the method of cognitive interviews also has certain limitations in terms of understanding and explaining students’ reasoning processes during task-solving, for instance due to a bias of social desirability (e.g., Kahne and Bowyer, 2017) as mentioned above or limited mental recall capacities. However, one central focus of the presented study lies on the investigation of self-awareness of one’s owns beliefs, i.e., whether the students were aware of their beliefs and whether they were aware if their beliefs influencing their perception, evaluation, selection and use of the given information. Hence, cognitive interviews were necessary to gain indications regarding the students (critical) reflection on their thought processes involved in solving the task, i.e., writing a statement. Especially any conclusions about self-awareness regarding one’s beliefs and their relation to decision-making can best be reached by means of stimulated recalls in cognitive interviews, which has been shown to approximate think-aloud methods in the study settings where participants cannot think aloud while processing the task (as in this computer-based test environment).

Follow-up research observing these limitations and implications would provide a better understanding of successful CR and a more significant basis for developing targeted instructional interventions in order to promote students’ CR skills in dealing with new more or less trustworthy or contradictory information.

## Data Availability Statement

The datasets presented in this article will be made available by the authors, without undue reservation, to any qualified researcher. Requests to access the datasets should be directed to troitschanskaia@uni-mainz.de.

## Ethics Statement

Ethical review and approval was not required for the study on human participants in accordance with the local legislation and institutional requirements. The participants provided their written informed consent to participate in this study.

## Author Contributions

OZ-T provided the idea for the study, co-developed the assessment, supervised the analyses, and co-wrote the manuscript. KB co-developed the assessment, supervised the analyses, and was involved in preparing and reviewing the manuscript. JF and DB conducted the analyses, and were involved in preparing the manuscript. SS was involved in the data collection and in the analyses. RS was involved in the development of the performance assessment and in preparing the manuscript. All the authors contributed to the article and approved the submitted version.

## Conflict of Interest

The authors declare that the research was conducted in the absence of any commercial or financial relationships that could be construed as a potential conflict of interest.

## Footnotes

1 In contrast to other concepts related to *critical thinking*, critical online reasoning (COR) is explicitly limited to the online information environment and includes the specific ability of “online information acquisition”. While there is currently no unified definition of COR, there are numerous definitions of its related construct critical thinking (CT) that include and describe different dimensions or levels. For instance, Oser and Biedermann (2020) distinguish between CT as alertness, CT as immediate reflection, and CT as analysis. Facione(2004, p. 9) describes CT as “inference, explanation, interpretation, evaluation, analysis, self-regulation” (for further definitions of CT, see Moore, 2013; Beck, 2020). As Brookfield (1987) emphasizes, “Being a critical thinker involves more than cognitive activities such as logical reasoning or scrutinizing arguments for assertions unsupported by empirical evidence. Thinking critically involves us *recognizing the assumptions underlying our beliefs and behaviors*”.

2 One well-known test of this kind is the Watson-Glaser Critical Thinking Appraisal (2002), which comprises tasks on inferences, recognition of assumptions, deduction, interpretation, and evaluation of arguments (Watson and Glaser, 2002).

3 https://ktl.jyu.fi/fi/hankkeet/kappas/copy_of_lyhyesti

4 www.ets.org/heighten/about/critical_thinking/

5 http://practice.cappassessments.com

6 The criteria for selection from the overall sample and inclusion were the participants’ socio-biographical and educational characteristics to ensure: (1) gender balance, (2) age distribution, (3) course of study representation (all), (4) study year/progress (advanced students), and (5) prior education (e.g., completed vocational training).

7 The deviation from the total sample size (30 participants) is due to missing values.
